# Control over the strength of connections between modules: a double dissociation between stimulus format and task revealed by Granger causality mapping in fMRI

**DOI:** 10.3389/fpsyg.2015.00321

**Published:** 2015-03-27

**Authors:** Britt Anderson, Sherif Soliman, Shannon O’Malley, James Danckert, Derek Besner

**Affiliations:** ^1^Department of Psychology, University of WaterlooWaterloo, ON, Canada; ^2^Psychology Department, McMaster UniversityHamilton, ON, Canada

**Keywords:** task set, fMRI, Granger causality, reading, parity, numerical cognition, rostromedial frontal cortex

## Abstract

Drawing on theoretical and computational work with the localist dual route reading model and results from behavioral studies, Besner et al. (2011) proposed that the ability to perform tasks that require overriding stimulus-specific defaults (e.g., semantics when naming Arabic numerals, and phonology when evaluating the parity of number words) necessitate the ability to modulate the strength of connections between cognitive modules for lexical representation, semantics, and phonology on a task- and stimulus-specific basis. We used functional magnetic resonance imaging to evaluate this account by assessing changes in functional connectivity while participants performed tasks that did and did not require such stimulus-task default overrides. The occipital region showing the greatest modulation of BOLD signal strength for the two stimulus types was used as the seed region for Granger causality mapping (GCM). Our GCM analysis revealed a region of rostromedial frontal cortex with a crossover interaction. When participants performed tasks that required overriding stimulus type defaults (i.e., parity judgments of number words and naming Arabic numerals) functional connectivity between the occipital region and rostromedial frontal cortex was present. Statistically significant functional connectivity was absent when the tasks were the default for the stimulus type (i.e., parity judgments of Arabic numerals and reading number words). This frontal region (BA 10) has previously been shown to be involved in goal-directed behavior and maintenance of a specific task set. We conclude that overriding stimulus-task defaults requires a modulation of connection strengths between cognitive modules and that the override mechanism predicted from cognitive theory is instantiated by frontal modulation of neural activity of brain regions specialized for sensory processing.

## Introduction

**FIGURE 1:**
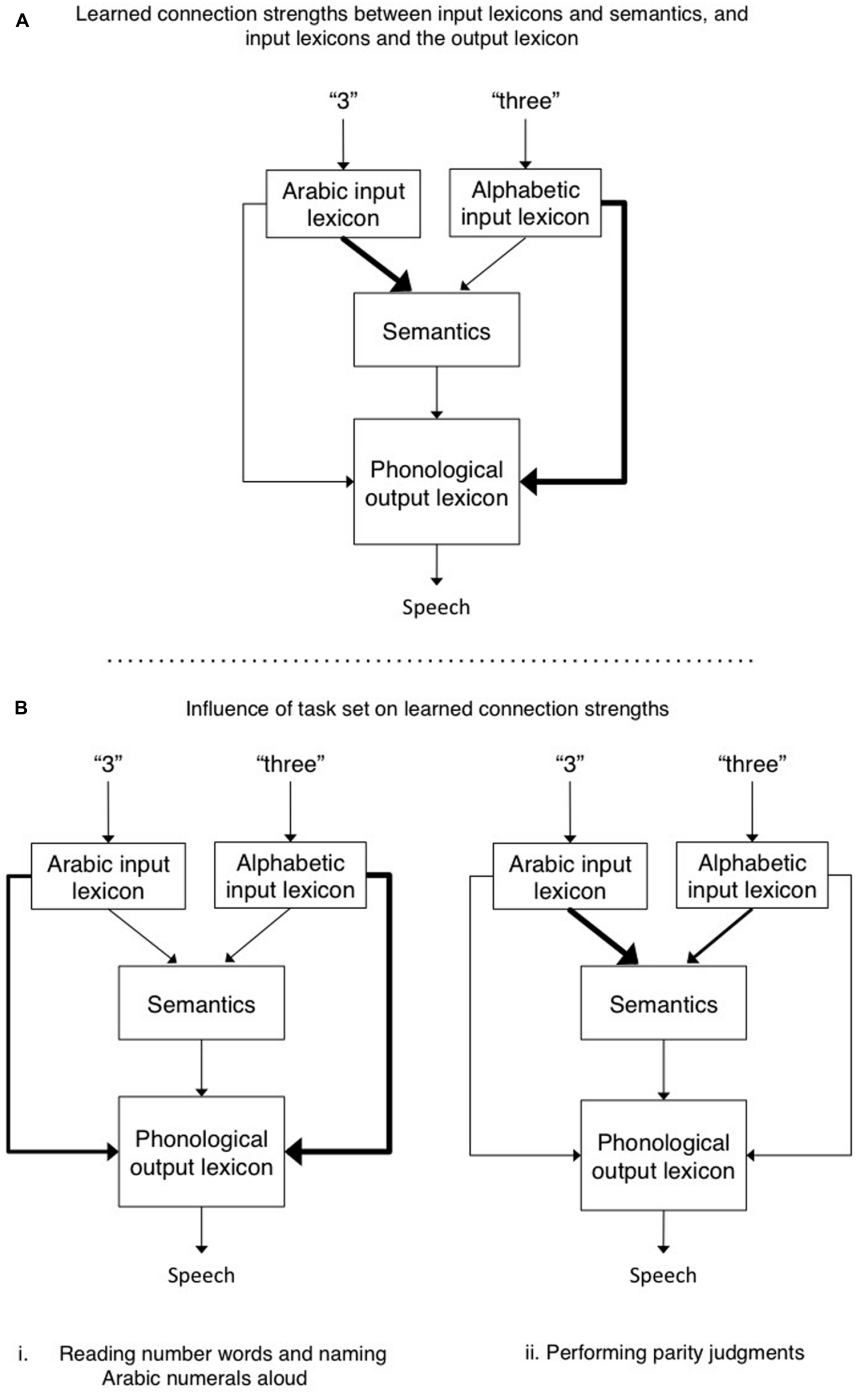
**(A)** The default connection strengths between Arabic and alphabetic input lexicons and semantics, and between the input lexicons and the phonological output lexicon. Performing parity judgment requires access to semantics to evaluate whether the number is odd or even. In contrast, when reading a number word aloud or naming an Arabic numeral aloud, the direct connection between their respective input lexicons and the phonological output lexicon suffice. Thicker lines reflect stronger connections between modules. **(B)** The hypothetical role of task set is to increase and/or decrease connection strengths between various modules so as to over-ride the default connection strengths. For example, in B. (ii), the connection strength from the alphabetic input lexicon to semantics is increased when making parity judgments to number words, and at the same time the strength of connections from alphabetic input lexicon to output phonology is decreased so as to reduce competition between these two routes.

The chain from sensation to perception and action is forged by context, and therefore, experience predisposes us to interpret certain stimuli in particular ways. In response to task demands we can overcome these biases, but the predispositions for action are still revealed by the time it takes us to act. As an example, when shown the word “three” we can either read it aloud or report its parity, and we can do the same when shown the numeral “3.” It turns out that the time to respond interacts with the nature of the format and reveals the omnipresent effect of a stimulus-task predisposition. Besner et al. (2011) found that participants were much faster at making parity judgments to Arabic numerals than to numbers presented alphabetically (hereafter referred to as “number words”), but took about the same amount of time when reading/naming these different stimuli aloud^1^. To explain their findings, Besner et al. (2011) proposed a general account in which there are various special purpose modules, each of which computes specific information-processing routines. An example of such an account can be seen in **Figure 1**, which is adapted in large part from several well known and highly successful computational accounts of reading aloud (e.g., Coltheart et al., 2001; Perry et al., 2007). The lexical representations are localist and linguistic in nature. Each node in the alphabetic input lexicon consists of the spelling of a word known to the reader. The nodes in the Arabic input lexicon represent, minimally, each single Arabic numeral from 0 to 9. The semantic system is conceptual and contains general knowledge of the world. Both input lexicons and the semantic system also activate the phonological output lexicon, which contains information about how each item the reader knows should be pronounced. The additional but theoretically central point here is that the strength of the format-task associations reflects both predispositions (some format-task pairings are more natural and experienced than others; **Figure 1A**), and task-induced modulation of the routes connecting the relevant cognitive modules (**Figure 1B**).

This account differs from previous accounts in its emphasis on the assertion that the connections between different representations differ in strength. The Arabic numeral format (an input module) is more strongly associated with semantics than is the number word format (hence parity judgments are faster for the former than the latter). In contrast, the number word format (input module) is more strongly associated with phonology, as compared to Arabic numerals. Indeed, so strongly that it overcomes the fact that Arabic numerals are more frequently encountered in the world than are their number word counterparts (and hence Arabic numerals, despite the fact that they are more frequent than their number word counterparts, are not named faster). Such a framework implies the need for some sort of mediator that functions as an arbiter of task demands. Such task demand units (e.g., see the seminal paper by Cohen et al., 1990) could favor some modules over others (e.g., by inhibition and/or activation), and/or modulate the strength of connections between different modules (see also Norman and Shallice, 1986; Monsell and Driver, 2000; Kane and Engle, 2003). Seeking converging evidence to support this hypothesis, the present work assessed whether this cognitive account has a neurological correlate by measuring changes in functional connectivity in functional magnetic resonance imaging (fMRI) scans of participants engaged in our reading/parity tasks with number words and Arabic numeral stimuli.

### Choosing a Functional Analysis Methodology

Earlier we proposed that there must be changes in the connection strength between modules that are modulated by combinations of task demands and stimulus characteristics (Besner et al., 2011). To provide evidence for our claim, we repeated our behavioral task while performing fMRI. We were specifically interested in whether we could observe changes in cortical functional connectivity for particular task-stimulus combinations. While the cognitive model *per se* is agnostic about underlying cortical anatomical localization, the claim about changes in connectivity is more generic. As long as the brain is the physical substrate implementing the cognitive components, functional changes in cognitive module connectivity should produce some corresponding changes in brain signals. The key question is whether the brain changes occur for particular task-stimulus set combinations that are predicted by the cognitive model, rather than whether the brain changes occur in particular brain regions. However, if changes are found, the knowledge about the localization of cognitive functions from prior functional imaging work does provide a powerful check on the plausibility that the changes in functional connectivity are meaningful.

Functional connectivity assessments in fMRI are relatively new, and there are several available procedures, and no one method has been clearly established as superior to the others (Rogers et al., 2007). Therefore, the choice of which one is used rests to a large extent on the familiarity and experience of the researchers involved. Perhaps the only real choice is whether there is sufficient *a priori* information available to warrant establishing a causal model in advance of experimental measurements. If not, and the experiment is largely exploratory then most of the available methods could be justified, though as will be reviewed shortly, only the Granger metric offers the potential for supporting a claim about the causal nature of the relation between two brain regions.

Essentially, functional connectivity is a way of measuring to what degree different voxels from a series of functional images move in sync together. Procedures for measuring this type of functional connectivity include GCM, psychophysiological interaction (PPI), and various graph theoretic measures. The exception to this approach may be dynamic causal modeling (DCM) where the emphasis is more on comparing particular models of brain module connectivity than exploring what voxels show activation patterns that are correlated.

One of the more direct approaches to functional connectivity is the graph theoretic (Cao et al., 2014). For this technique (Bullmore and Bassett, 2011) the BOLD signal for each voxel can be assessed over time. These time series are then correlated against each other to see which voxels are correlated with which other voxels. Of course, adjacent voxels are likely to show high correlations, and therefore it is more customary to first define particular brain nodes and to assess the correlation between nodes. From this large correlation matrix a threshold is selected, and all pairs of nodes showing correlations greater than the threshold are coded as one and the remainder as zero. This produces the connectivity graph from which common graph theoretic quantities (Rubinov and Sporns, 2010) may be computed. Graph theoretic measures of connectivity have been used for defining multimode networks and to compare the metrics of these networks across conditions.

Another correlation-based method is PPI. In PPI a seed region is selected, typically on the basis of this region showing some changes in activation as a function of task or condition. Then the time series of this voxel is extracted and the mean is subtracted. A dummy variable for the task conditions of interest is constructed and convolved with a hemodynamic response function, and this is also demeaned. Then the point-wise combination of these time series is constructed and used as a regressor in a conventional regression analysis. The effect of this point-wise product of the seed region time series and the task time series is to produce a time series where some epochs are anti-correlated to the task, and others are positively correlated. Voxels where this product regressor shows an interaction will identify other voxels that are functionally connected to the seed region and are modulated by task (O’Reilly et al., 2012). This procedure does not measure if the relations are causal, but only if there are significant interactions. In common with all the functional connectivity procedures there is the challenge of low power and of spurious correlations. The latter emerges because there may be common linkages that we are not aware of, and also because of the large number of shared variables that influence BOLD measurements across time and voxels.

Unlike the two methods just considered, the next two methods are potentially able to make causal assertions about the nature of the relations between brain regions and functional measurements of voxels (which is not limited to fMRI, but can also be applied to other imaging methodologies as well as electrophysiology measures). DCM is a general approach that comes in different varieties. The common element in DCM is that specific models are constructed before data collection, and then the different models are compared for their ability to explain the observed activation patterns. Typically this procedure involves a specification of hidden neural dynamics, often with differential equations, that are linked by other equations to predictions for measured data. The probability of observing the data given the models are fit with Bayesian inversion principles and then model selection principles (Stephan and Roebroeck, 2012) are used to pick the best of the *a priori* models.

Granger causality mapping is another measure of effective connectivity. Unlike DCM it is best considered as a method of exploration rather than confirmation (Bressler and Seth, 2011). While graph theoretic analyses and PPI are correlative, GCM was developed with the idea of an autoregressive model, though this is not an obligatory component of statistical tests for GCM. However, the autoregressive origins of GCM give the key insight. GCM relies on the fact that causes precede consequences. If we are to compare two variables *X* and *Y*, and we can better predict *X* from knowledge of *Y* (even when we use all prior observations of X in our model), then we can assert that *Y* is Granger causal of *X* (Stephan and Roebroeck, 2012). According to these authors, true functional connectivity measures are correlative and “…a careful interrogation of temporal order structure in fMRI data should start (but not end) with looking for experimentally induced changes in the detected G-causality” (Stephan and Roebroeck, 2012, pg 859).

For our analysis we used GCM as our measure of connectivity because it was the method best known to us, we had an established fMRI tool box for its calculation, we could in principle (though we did not in practice) establish a causal direction for any functional connections found, and we were engaged in an exploratory study, a role for which GCM is thought to be well suited. We further explore the results of our GCM analysis in the Section “Results and Discussion.”

In summary, to examine our hypothesis that task specific contexts modulate connection strengths between cognitive modules we performed an fMRI experiment where participants performed a behavioral task that required them to either name/read Arabic numerals/number words or report the parity of these same stimuli. Our critical analysis examined whether there were changes in the strength of functional cortical connectivity as a function of task~stimuli conjunctions.

## Materials and Methods

### Subjects

Fifteen neurologically normal participants (eight female; average age = 24.8, SD = 2.4; 11 right-handed) with normal or corrected to normal vision were paid $50 to participate in the fMRI session at Grand River Hospital in Kitchener, ON, Canada. Thirteen of these participants were paid an additional $5 to complete a subsequent behavioral session in which response times (RTs) and errors were recorded (two could not participate due to scheduling difficulties). All participants completed the fMRI session before completing the behavioral session. Informed consent was obtained from each participant, prior to each session. The Office of Research Ethics, University of Waterloo and Tri-Hospital Research Ethics Board of Waterloo, ON, Canada approved the study.

### Study Design

**FIGURE 2:**
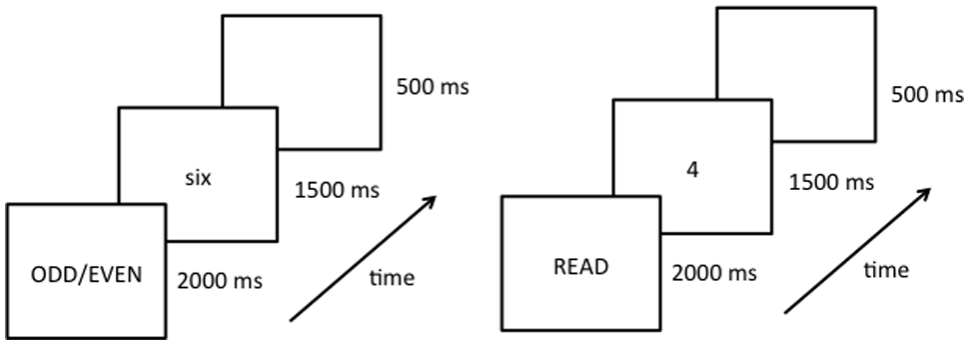
Schematic diagram of the experimental procedure showing the instruction slide (instructing participants to either read or perform parity judgments) and an example of the first trial of a given block. Each run starts with a fixation symbol (lasting 16 s), followed by 16 blocks of experimental trials (each block lasting 18 s, and displaying either number words or Arabic numerals for the entire block), ending with another fixation symbol (16 s).

During the fMRI session, stimulus presentation was controlled by a Dell laptop computer running E-Prime software (Version 1.1; Schneider et al., 2002). Two behavioral tasks were used within a block design (**Figure 2**). Each block consisted of eight trials lasting 2000 ms per trial. Prior to each block of trials an instruction message, either the word “READ” or “ODD/EVEN,” was presented for 2000 ms. Prior to the study, participants were instructed to perform the given task silently on all subsequent trials until they saw the next instruction slide. Specifically, the researcher instructed them that “when the task is to read, think the number word silently in your head, and when the task is parity think the correct word (odd or even) silently in your head.” On each trial a number word or Arabic numeral appeared in the center of the screen for 1500 ms, followed by a 500 ms blank screen. Within each block the numbers 2 through 9 appeared in random order. Stimulus type (number words or Arabic numerals) was held constant within each block. Each experimental run began and ended with a 16 s fixation epoch. Within a single experimental run there were 16 task epochs lasting 18 s each for a total run length of approximately 5.5 min. Within each run, task (reading/naming or parity) and stimulus type (number words or Arabic numerals) was repeated four times (**Figure 2**). Each participant completed four experimental runs in which the order of the tasks and stimulus type was different.

During the behavioral session vocal responses were collected using a Plantronics (Santa Cruz, CA, USA) LS1 microphone headset and a voice-key assembly. The study design was the same as the fMRI session with the following exceptions: first, the fixation symbols that began and ended each run were presented for only 2 s (because no baseline rest period was needed during this session). Second, participants were to say all responses aloud, into a microphone, and a blank screen replaced the target once the voice-key registered a response. Finally, after each trial, the experimenter coded responses as correct, incorrect, or mistrial (mistrials represent voice-key errors, or extraneous background noise). Once the response was keyed in, the blank screen remained for another 100 ms, at which time the next trial began.

### fMRI Data Collection

Functional data were collected using gradient echo-planar T2^∗^-weighted images acquired on a Philips 1.5 Tesla machine (TR = 2000 ms; TE = 40 ms; slice thickness = 5 mm with no gap, 26 slices; FOV = 220mm × 220 mm; voxel size = 2.75 mm × 2.75 mm × 5 mm; flip angle = 90°). An experimental run consisted of 26 slices/volume, 160 volumes, eight per fixation baseline at the beginning and end of each run, and nine per block for the four experimental conditions (each repeated four times in a single run). At the beginning of the session, a whole-brain T1-weighted anatomical image was collected for each participant (TR = 7.5 ms; TE = 3.4 ms; voxel size = 1 mm × 1 mm × 1 mm; FOV = 240 × 240 mm; 150 slices; no gap; flip angle, 8°). Stimuli were presented on an Avotec Silent Vision (Model SV-7021) fiber-optic visual presentation system with binocular projection glasses controlled by a Dell laptop running E-Prime software synchronized to trigger-pulses from the magnet.

### fMRI Data Processing

Imaging data were analyzed using Brain Voyager QX (version v2.4.2.2070, Brain Innovation B. V., Maastricht, The Netherlands). Anatomical images were transformed to Talairach coordinates with a voxel resolution of 1 mm × 1 mm × 1 mm using sinc interpolation (Talairach and Tournoux, 1988). Each functional run was visually inspected for motion artifacts by playing a virtual movie of each functional volume in sequence (Culham et al., 2003). Trilinear interpolation was used to correct for motion artifacts in all functional runs and data were pre-processed, which included slice-time correction, linear trend removal, and three cycles of temporal high pass filtering. Functional images underwent 3D motion correction and temporal filtering using a frequency-space filter (FFT) with a cut-off of three cycles. No spatial or temporal smoothing was applied to the data.

### Data Analysis

The hemodynamic response function for each functional run was convolved to the Boynton HRF. Next, four linear predictors of the BOLD response were created for each participant, corresponding to the four tasks (read number words, read Arabic numerals, parity judgments on number words, parity judgments on Arabic numerals). These 60 linear predictors (15 participants × 4 predictors each) were then entered into a random effects general linear model. GCM (GCMPlugin **1.5** for Brain Voyager v2.4.2.2070; Roebroeck et al., 2005) was used to examine the functional connectivity related to the different tasks and stimuli. All reported significance values were Bonferroni corrected for multiple comparisons. Seed areas for GCM were chosen for each of the principal task and stimulus selective regions. Ultimately, the largest seed region, and the one that produced theoretically important results was the left medial occipito-temporal area. We discuss GCM and our rationale for choosing the seed region in detail in the relevant results section below.

## Results

### Behavioral Results

Mean RTs were entered into a 2 (naming/reading-aloud task vs. parity task) × 2 (Arabic numerals vs. number words) ANOVA. All factors were within subjects. Mean RTs and percentage errors for each condition can be seen in **Table 1**. Errors (0.7% of all responses) and mistrials (4.2%) were removed prior to the RT analysis. Given the very small number of errors, we did not analyze them further. RTs for the naming/reading-aloud task were faster than RTs for the parity task, *F*(1,12) = 73.2, MSE = 4669.5, *p* < 0.001. There was also a main effect of stimulus type in which Arabic numerals were responded to faster than number words, *F*(1,12) = 21.1, MSE = 164.7, *p* < 0.01. Critically, we replicate the pattern reported by Besner et al. (2011) in that there was a significant interaction between task and stimulus type, *F*(1,12) = 53.2, MSE = 141.1, *p* < 0.001. The mean RTs in **Table 1** show that the interaction between task and stimulus type reflects the fact that when performing parity judgments responses are faster to Arabic numerals than to number words, *t*(12) = 6.94, *p* < 0.001, but when reading aloud the number words are read aloud slightly (but only marginally significantly) faster than the Arabic numerals are named, *t*(12) = 2.12, *p* < 0.056^2^.

**Table 1:** Mean RTs (ms) and percentage errors (%E) in the naming/reading aloud and parity tasks as a function of presentation format.

	RT		%E	
	Parity	Reading aloud	Parity	Reading aloud
Number words	686	500	1.2	0.4
Arabic numerals	645	507	0.7	0.3
**Difference**	**41**	**-7**	**0.5**	**0.1**

### fMRI Results

#### Task and Stimuli Contrasts

We first addressed the question of which brain areas were associated with main effects of task (reading/naming or parity judgment) and stimuli (Arabic numerals or number words). We examined the main effects of task and stimulus type and their interaction and found significant activation in a total of eight brain regions. Talairach coordinates and cluster sizes of these regions can be seen in **Table 2**. Significantly higher activation for number words vs. Arabic numerals was observed in the left and right lingual gyri (BA 17/18), and in the left fusiform area (BA 37). The reverse contrast (Arabic numerals > number words) found no areas of significant activation.

**Table 2:** Results of overall fMRI brain analysis.

Region	Hemisphere	Talairach coordinates			BA	Threshold	Cluster size
		x	y	z			
**Number words > Arabic numerals**							
Lingual gyrus	L	-17	-88	-13	17/18	*p* < 0.0001	17,171
	R	17	-86	-13	17/18	*p* < 0.0001	11,348
Fusiform	L	-40	-48	-24	37	*p* < 0.0001	2,698
**Reading > parity**							
Superior occipital	L	-40	-81	22	19	*p* < 0.0001	789
**Parity > reading**							
Medial frontal	L	-5	2	53	6	*p* < 0.0001	1,565
Precentral gyrus	L	-50	-3	-43	6	*p* < 0.0001	283
Inferior parietal	L	-47	-40	40	40	*p* < 0.0001	992
Cerebellum	R	33	-59	-26		*p* < 0.0001	1,768

When contrasting activation for reading/naming vs. parity judgments (reading/naming > parity), increased BOLD activity was observed in the left superior occipital region (BA 19). The reverse contrast (parity > reading/naming) revealed significant activation in the left medial frontal area (BA 6), left precentral gyrus (BA 6), the left inferior parietal area (BA 40), and the right cerebellum. Finally, we found no regions of increased BOLD signal for the interaction term.

#### Granger Causality Modeling

In our introduction we explained our reasoning for choosing GCM as our method for examining functional connectivity in the brain. Now we would like to explain GCM itself in more detail. GCM is a method for determining the direction of causal relations (Granger, 1969) between time series data. A time series is essentially a list of data where each element in the list is indexed by a time, (e.g., the daily high temperatures recorded by weather stations, or the BOLD signal measured from an MRI voxel across a sequence of functional images). Given two such lists of BOLD activity and their times of occurrence, GCM represents one approach for determining whether there is a functional relation between them, and possibly also a direction of causal influence. The logic can be demonstrated through the use of multiple regression analysis. Imagine that we have one voxel labeled A, and another labeled B. For each of them, A and B, we can designate the value at a particular time with A(*t*) or B(*t*). Earlier times would be notated as A(*t*-1) or B(*t*-10) for example. We can imagine that if there is some autocorrelation then earlier values of A would predict later values of A. We could quantify this relationship by constructing a regression model in which we compute the statistical relation between A(t) and earlier times as A(*t*) ~ A(*t*-1) + A(*t*-2) + … + A(*t*-*n*) for some *n* that we believe, or can demonstrate, is as large as necessary to contain the period of self-influence. If we then fit another regression model A(*t*) ~ A(*t*-1) + … + A(*t*-*n*) + B(*t*′) and it provides a statistically significant improvement in fit, then we know that B is related to A. If *t*′ is equal to some earlier point in time, (e.g., *t*-1), then we can say that B is “Granger Causal” of A. If instead, it is earlier times of A that predict something about B not obtainable from earlier times in the B time series then we can say that A is “Granger Causal” of B. If *t*′ is equal to *t* then B is, in the language of fMRI, “functionally connected” to A and the direction of causality is unknown. Because of the relatively slow nature of the hemodynamic response function, this result is not uncommon.

Granger causality is a theory of binary relations. It does not exclude the possibility of a third location causally affecting both of the studied time series (A and B in the example above). This is of concern in fMRI because many factors may have joint effects on multiple voxels, perhaps with a fixed temporal offset. Thus, finding a Granger causal relation is easier to interpret when the degree of relation varies across experimental conditions. This makes it unlikely that unknown mechanical or physiological factors having a common effect throughout an MRI acquisition session can explain the presence of the functional or causal relations.

In the present study, seed areas for GCM were chosen based on the thresholds and cluster sizes of activated regions. GCM was performed on the pooled data for all participants subdivided by the four stimulus-task combinations. The threshold was increased until the activation areas were limited to relatively large cluster sizes of 100–300 voxels (**Table 2**), to ensure that an area that barely met the threshold was not chosen over an area with greater activation. The above process resulted in three candidate seed regions: a cluster of 189 voxels in the left frontal region at a threshold of *p* < 0.0001, a cluster of 235 voxels in the left occipito-temporal region at a threshold above the maximum of *p* < 0.0001, and a cluster of 137 voxels in the left parietal region at a threshold of *p* < 0.0001^3^.

**FIGURE 3:**
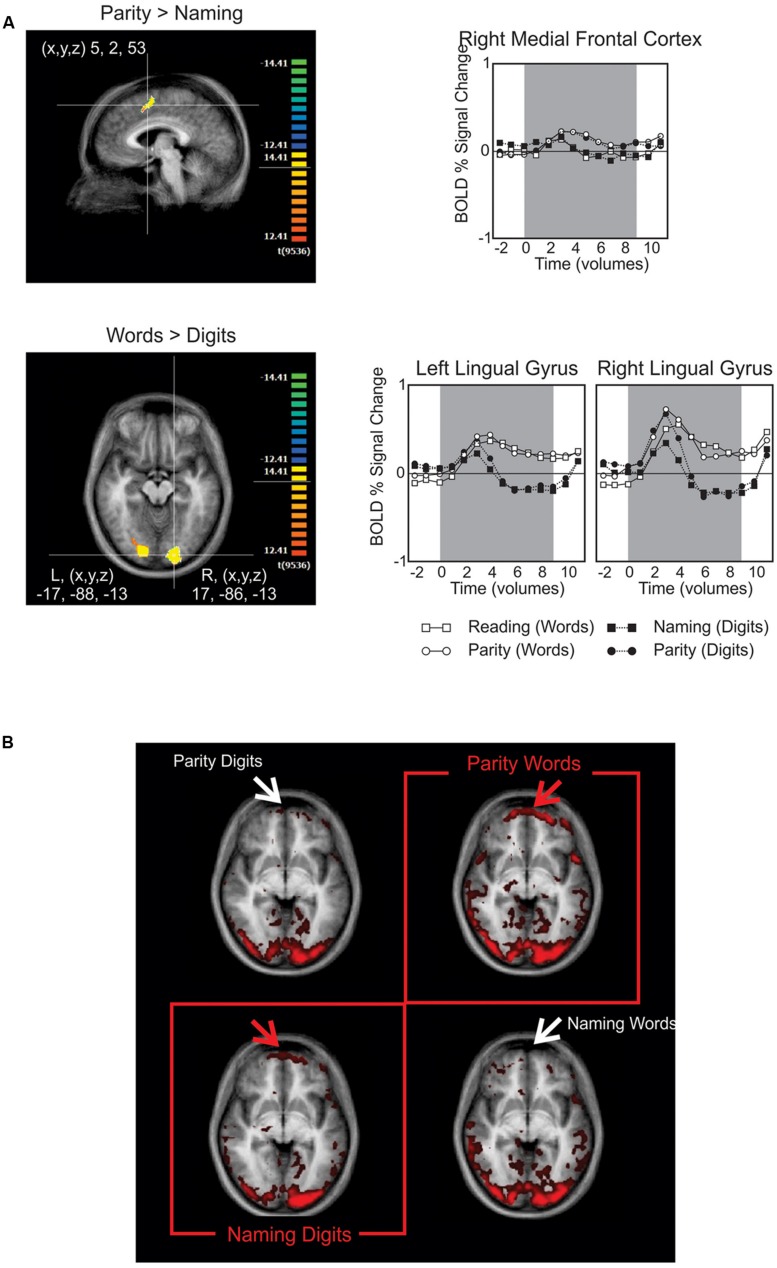
**(A)** BOLD signal contrasts. The upper panel shows medial frontal activity when contrasting parity > reading. The lower panel highlights the posterior activity found for contrasting number words > Arabic numerals, which was later used as the seed region for the Granger causality mapping (GCM). The graphs to their right provide the time course for BOLD signals in these regions subdivided by task (upper) or stimulus type (lower). **(B)** In this GCM correlation map, the red and white arrows point out the presence and absence of activation in the fronto-polar cortex, respectively. The red boxes highlight the resulting dissociation, in which frontal activation is only present when performing the non-default tasks: naming Arabic numerals and performing parity judgments on number words.

The only seed region from the above candidates that produced any simultaneous association of interest in the GCM was the left occipito-temporal region identified from contrasting the presentation of number words with Arabic numerals (**Figure 3A**). Robust Granger causal relations were identified by the GCM procedure between this seed region and an anterior frontal region, but only when the time series were contemporaneous (aka “instantaneous”; Goebel et al., 2006). That is, we did not observe any robust associations when the time series were lagged to precede or succeed one another. Nevertheless, setting the left medial occipito-temporal region as the seed region for the GCM yielded a theoretically important dissociation. As demonstrated in the instantaneous GCM in **Figure 3B**, there is a strong association for BA 10 (fronto-polar cortex), predominantly at the frontal polar region, extending more medially than laterally, and the occipital temporal seed region only when naming Arabic numerals or performing parity judgments on number words.

One limitation of our procedure, and in keeping with the exploratory role for GCM, is that we cannot do an “interaction” analysis as one might do with an ANOVA procedure to demonstrate the differences seen across conditions is “statistically significant.” Given the association found here, future studies could use DCM methods to statistically compare models that include a contextually modifiable connection between BA 10 and inferior occipital-temporal regions to models that do not.

## Discussion

We have previously proposed that the interaction between tasks and stimulus format observed in behavioral experiments can be understood in terms of differences in stimulus-specific module connectivity (Besner et al., 2011). In this paper we explored whether fMRI can be a useful adjunctive source of information for evaluating this cognitive account.

We assert that fMRI can be used analogously to RT data for evaluating cognitive theories. The assumption underlying the use of RT data for evaluating cognitive accounts is that a change in RT reflects a change in some aspect of cognitive operations. Our claim here is that changes in the magnitude and patterns of cerebral metabolic activity recorded with fMRI are another source of such data. In addition, because functional roles have been established for many brain regions, the specific brain region indicated by an fMRI study and its functional associations can be used to evaluate the plausibility of a particular cognitive account. In the present study we replicated, at the behavioral level, what we reported previously: parity judgments to Arabic numerals are much faster than parity judgments for number words, but this difference is eliminated when reading/naming aloud. This is consistent with our prior account of changes in the task driving changes in the strength of connectivity between modules so as to permit the correct task to be performed. We support this account with the central finding of our fMRI experiment. That is, that metabolic activity in BA 10 reveals stimulus-selective functional connectivity to posterior visual areas *only* when task demands *conflict* with the natural stimulus-task predispositions (i.e., conflict is present when naming Arabic numerals and making parity judgments to alphabetically presented number words). Specifically, despite finding no regions of increased BOLD signal for the interaction between task and stimuli, the important dissociation is present when examining the correlated activations of the fronto-polar cortex and the medial occipito-temporal region across task-stimulus combinations. As predicted by our task/stimulus-set hypothesis, this indicates that the difference in processing across task-stimulus combinations is not local to a specific brain region, rather it reflects differences in the connectivity between regions as a function of task context. We found a frontal region (BA 10) that showed a significant increase in functional connectivity as measured by GCM only for the two combinations of stimulus type and response mode that violated “natural” (learned) predispositions. That is, stronger functional connectivity between BA 10 and the medial occipito-temporal region is seen when the participant must inhibit the tendency to read aloud the number words in order to make parity judgments of them, and conversely, when the participant has to inhibit the tendency to activate semantic (parity) information in order to name Arabic numerals. Such frontal activation is not seen when reading aloud number words and making parity judgments to Arabic numerals (by hypothesis, the stronger and more natural associations).

The above finding is even stronger given prior knowledge of the roles of the fronto-polar cortex, and BA 10, in the maintenance of a task set. The fronto-polar region, including BA 10, shows a pattern of activation consistent with the role of overriding a natural predisposition so as to permit a different task (dictated by the instructions or situational context) to be engaged (Koechlin and Hyafil, 2007; see also the task demand unit account proposed by Cohen et al., 1990). In a study that examined the neural involvement in exercising endogenous control in a task-switching context, Forstmann et al. (2005) found significant involvement of the fronto-polar area and the inferior frontal gyrus when participants needed to follow endogenous cues that were directly related to the task set and to transitioning from one task to another. Similarly, Koechlin et al. (2000) found that BA 10 was involved in processing both endogenous and exogenous plans, and that areas in the fronto-polar prefrontal cortex were “selectively activated when subjects have to keep in mind a main goal while performing concurrent (sub)goals” (Koechlin et al., 1999, p. 148).

The finding of BA 10 modulation by task demands also fits well with the model of rostral frontal cortex (BA 10) proposed by Burgess et al. (2007). This model was developed to account for the paradox that frontal activations are often seen for tasks that are relatively unaffected by frontal leisons. Burgess, Dumontheil, and Gilbert propose that BA 10 functions as a gateway for mediating the relative weighting of stimulus driven and stimulus independent access to central representations. One component of the proposal is a balancing of responses driven by sensory input and schema maintained by top–down control. The variation in GCM fits well with this model in that our tasks differ in the extent to which they are more and less “automatic” despite using the same physical stimuli. Another interesting connection to the Supervisory Attentional Gateway hypothesis is that this model has been explicitly connected to RT differences, which are an important component of the disassociation reported here.

In light of this existing knowledge regarding BA 10 and its involvement in goal-directed processing, task-maintenance, planning, and attentional gating we find the double-dissociation in BA 10 GCM, where it is only significant when there are complex task-stimulus type combinations, as corroborating evidence for a mechanism that controls and modulates action when the task set is in conflict with the default set.

In summary, we used GCM to examine functional connectivity in the brain analogous to that proposed by our cognitive account, and found changes in the coupling of frontal and posterior brain regions. These changes in coupling magnitude showed a qualitative pattern that complements our RT data. We conclude that stimulus-task interactions in single number word reading/Arabic numeral naming and parity judgments reflect dynamic differences in module connectivity. In particular, frontal activation is associated with control over holding the appropriate task set in mind so as to permit the correct response to be made despite competition from the learned associations between stimuli and psychological processes. With the prior findings of the role of BA 10, we take these the present results as support for our cognitive account that differing connection routes or connection strengths between cognitive modules are associated with the maintenance of a task set.

Finally, it is important to note how the present findings do not fall victim to the *consistency fallacy*: If an explanation takes consistency between obtained neuroimaging results and the predictions of a cognitive theory as corroboration of the cognitive theory, without enumerating other possible results that, if obtained, would have *falsified* the cognitive theory, the explanation commits a consistency fallacy (Coltheart, 2013). In the work reported here, any other possible pattern in the GCM analysis would have been *inconsistent* with the predictions of the task set account. The only pattern that supports the cognitive account is one in which the fronto-polar cortex is primarily active when the task conflicts with the default set (i.e., naming Arabic numerals and performing parity-judgments on number words). That is, it is logically possible that area BA 10 would have been active (a) in all combinations of task and default set, (b) in none of those combinations, (c) in only one of them, and so on. In any of those cases, the fMRI results would have run contrary to the cognitive account.

At the global level, we conclude that fMRI results, coupled with GCM, are another source of data, like RTs and errors, which can be usefully applied to the evaluation of cognitive accounts. At the local level, the fMRI results are consistent with an account in which the interaction between stimulus type and tasks can be understood as a reflection of differing connection strengths between various cognitive modules (the default set). Correct task performance in the current context depends upon coordinated frontal regulation that overrides the dominance of the default set so as to enable and maintain the appropriate task set.

## Conflict of Interest Statement

The authors declare that the research was conducted in the absence of any commercial or financial relationships that could be construed as a potential conflict of interest.

## Footnotes

1 Support for this strength of connections account has been reported by Adelman et al. (2014) who compared two versions of a computational model for reading words aloud. The model in which word frequency affects the strength of connections (stronger for high- than low-frequency words) between an orthographic input lexicon and a phonological output lexicon accounts for more variance in reading aloud response times than does the otherwise identical model in which word frequency affects the resting levels of activation (higher resting levels for high- than low-frequency words) in these same modules.

2 Damian (2004) reported a similar pattern of results, except that in his experiment, reading aloud number words was significantly faster than naming Arabic numerals.

3 The threshold surpassed the point at which the *p*-value gives a meaningful number (the last meaningful number is on the order of e-43, beyond that, 0.0000e + 00 is shown).
